# 3D Raman imaging of systemic endothelial dysfunction in the murine model of metastatic breast cancer

**DOI:** 10.1007/s00216-016-9436-9

**Published:** 2016-03-02

**Authors:** Marta Z. Pacia, Elzbieta Buczek, Agnieszka Blazejczyk, Aleksandra Gregorius, Joanna Wietrzyk, Stefan Chlopicki, Malgorzata Baranska, Agnieszka Kaczor

**Affiliations:** Faculty of Chemistry, Jagiellonian University, Ingardena 3, 30-060 Krakow, Poland; Jagiellonian Centre of Experimental Therapeutics (JCET), Jagiellonian University, Bobrzynskiego 14, 30-348 Krakow, Poland; Department of Experimental Oncology, Institute of Immunology and Experimental Therapy, Polish Academy of Sciences, R. Weigla 12, 53-114 Wroclaw, Poland; Department of Experimental Pharmacology, Jagiellonian University, Grzegorzecka 16, 31-531 Krakow, Poland

**Keywords:** 3D Raman imaging, Cancer metastasis, Endothelium, Ex vivo, Pathology marker, Protein overproduction

## Abstract

It was recently reported in the murine model of metastatic breast cancer (4T1) that tumor progression and development of metastasis is associated with systemic endothelial dysfunction characterized by impaired nitric oxide (NO) production. Using Raman 3D confocal imaging with the analysis of the individual layers of the vascular wall combined with AFM endothelial surface imaging, we demonstrated that metastasis-induced systemic endothelial dysfunction resulted in distinct chemical changes in the endothelium of the aorta. These changes, manifested as a significant increase in the protein content (18 %) and a slight decrease in the lipid content (4 %), were limited to the endothelium and did not occur in the deeper layers of the vascular wall. The altered lipid to protein ratio in the endothelium, although more pronounced in the fixed vascular wall, was also observed in the freshly isolated unfixed vascular wall samples in the aqueous environment (12 and 7 % change of protein and lipid content, respectively). Our results support the finding that the metastasis induces systemic endothelial dysfunction that may contribute to cancer progression.

**Electronic supplementary material:**

The online version of this article (doi:10.1007/s00216-016-9436-9) contains supplementary material, which is available to authorized users.

**Graphical Abstract:**
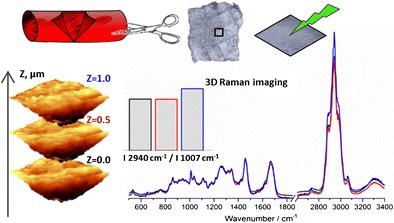
Schematic illustration of methodology of sample preparation and measurement

## Introduction

Breast cancer is an insidious disease that is often not diagnosed at the initial, but rather at the late stage, when metastases in lymph nodes and distant organs are already formed. The most common metastatic foci of breast cancer were detected in the lungs, brain, pleura, liver, and bones. As it was shown in the very recent work in *Nature* [1], the process of formation of metastatic sites is determined by tumor exosome integrins. The tumor-derived exosomes (e.g., lung–tropic exosome with special integrins) are directed toward specific organs and prepare the pre-metastatic niche after reaching the place of destination (e.g., in the lungs). It means that direction of formation of metastasis sites can be predicted based on integrins’ expression profiles of circulating plasma exosomes [1]. In order to form metastatic sites, migrating cancer cells have to overcome the blood–tissue barrier in the circulatory system, i.e., the endothelium. As long as the endothelial layer is healthy and continuous, it plays a crucial role in vascular repair and tumor invasiveness inhibition, while dysfunctional endothelium may act in the opposite way [2].

Raman spectroscopy is a label-free method providing chemically specific information about the studied sample [3]. Alterations occurring in tissues upon development of diseases cause changes in the concentration and structure of the biomolecules that is reflected in tissue Raman spectra. A Raman-based approach enables not only characterization of differences in the tissues of healthy and pathologic specimens, but also discrimination between the Raman spectra of normal and abnormal tissues [4] that establishes a base of medical diagnostics. Up to now [4–8], Raman studies on cancer concentrated on characterization of differences between cancerous or precancerous and control tissues.

The spectral features of cancerous murine and human tissues are remarkably similar. In the human breast tissues, the changes in the relative content of fatty acids, triglycerides, proteins, carotenoids and water were recognized as a hallmark of cancer [7]. The main components of noncancerous tissues were lipids with unsaturated fatty acids, while the Raman spectra of cancerous tissues were dominated by bands originating from proteins [5]. In the murine model of the breast cancer (BALB/c mice inoculated with 4T1 murine mammary tumor cell line), changes in the chemical composition in breast tissues [6, 8] and lymph nodes [8] were previously reported. The distinction between normal mammary tissues from mammary tumors was based on the decrease of lipid bands (1747 and 1302 cm^−1^) with the simultaneous increase of signals associated with proteins (1265, 1002, and 850 cm^−1^) [8]. Moreover, Raman spectroscopy enabled detection of tissues distinctly different from normal or tumor ones, i.e., tissues with early biochemical changes prior to definite morphologic tumor development [6].

To the best of our knowledge, the chemical changes occurring in the vascular wall along the progression of primary cancer and metastasis were not studied so far with the help of Raman spectroscopy. In this work, we aimed to analyze spectroscopic features of the vessel wall and particularly the endothelium, the barrier for migrating cancer cells that was recently proven to undergo significant alterations upon cancer metastasis [2, 9]. Raman imaging was previously demonstrated to be an effective method of studying endothelium status in the murine models of various diseases associated with endothelial dysfunction including diabetes [10], hypertension [11], and atherosclerosis [12]. In the present study, Raman confocal imaging was used to investigate the influence of the metastatic breast cancer on the endothelial layer of the murine aorta. We characterized the considerable chemical changes occurring in the aortic endothelium upon cancer-associated endothelial dysfunction. 3D Raman imaging enabled to demonstrate that biochemical tissue alterations were limited to the endothelial layer, rather than the whole vessel wall and, therefore, could be treated as a hallmark of endothelial dysfunction related to cancer-induced systemic inflammation.

## Materials and methods

### Murine model of metastatic breast cancer

Seven- to eight-week-old BALB/c female mice (obtained from Charles River Laboratories) were orthotopically inoculated into the right mammary fat pad with 1 × 10^4^ viable 4T1 tumor cells (obtained from American Type Culture Collection) cultured according to previously described protocol [9]. Healthy BALB/c mice were used as a control group. Analysis was conducted 6 weeks after cancer cell transplantation. Animals (*n* = 5 for both control mice and mice with the cancer metastasis) were anesthetized by an intraperitoneal injection (i.p.) of a mixture consisting of ketamine and xylazine (100 mg ketamine/10 mg xylazine/kg body weight). The chest was opened and the thoracic aorta was quickly removed and transferred into Krebs–Henseleit buffer.

All experimental procedures involving animals were conducted according to the Guidelines for Animal Care and Treatment of the European Communities and the Guide for the Care and Use of Laboratory Animals published by the US National Institutes of Health (NIH Publication No. 85-23, revised 1996). All procedures were approved by the Local Ethical Committee on Animal Experiments.

### Preparation of fixed and unfixed aorta

For fixed tissue preparation, the resected and split-open arteries were tightly glued to the Cell-Tak®-coated calcium fluoride surface in air. Subsequently, the tissue was preserved by a 10-min soak in 4 % buffered formalin and twice flushed with distilled water. The samples were measured after drying in air.

For unfixed sample preparation, the fragments of the aorta were attached on Cell-Tak®-coated Petri dish and kept in minimal essential medium (MEM) with the addition of 1 % MEM vitamins, 1 % antibiotics (penicillin 10,000 U/mL and streptomycin 10,000 μg/mL), 1 % non-essential amino acids (NEAA), and 20 % fetal bovine serum. Aorta preparations were incubated at 37 °C and 5 % CO_2_ for at least 12 h. Shortly before measurements, the samples were flushed twice with isotonic phosphate buffer (PBS) at 37 °C and stored in it during data acquisition. The temperature during measurements was about 23 °C.

### Instrumentation

Raman imaging was done with a Confocal Raman Imaging system WITec alpha 300 with the application of a ×100 air objective (Olympus, MPlan FL N, NA = 0.9) and ×60 water immersion objective (Nikon Fluor, NA = 1) for fixed and unfixed tissues, respectively. The laser excitation wavelength of 532 nm, laser power of ca. 10 and 20 mW, and the integration time of 0.2 and 0.3 s/spectrum were used in all cases for fixed and unfixed tissues, respectively. For both preparation methods, images of the edge length of 15 × 15 μm^2^ (75 × 75 pixels^2^) were recorded. The spectral resolution was 3 cm^−1^.

Subsequently after Raman measurements, AFM imaging (Confocal Raman Imaging system WITec alpha 300) in the pulsed force mode (PFM) with the force modulation probes (*k* = 2.8 N/m, WITec) was performed for fixed tissues. The resolution of images was 200 × 200 pixels^2^ for the area of 20 × 20 μm^2^.

WITec Project Plus 2.10 software was employed for data preprocessing including cosmic ray removal. All spectra were baseline corrected using a polynomial or autopolynomial function of degree 2 for fixed and unfixed samples of the vessel wall, respectively. The OPUS 7.2 program was used for the vector normalization of the spectra (in the 3200–450 cm^−1^ and 1500–200 cm^−1^ regions, for fixed and unfixed samples, respectively), and calculations of the integral intensity of the following bands: 2940 cm^−1^ (in the regions: 3030–2825 cm^−1^ and 3012–2820 cm^−1^ for fixed and unfixed tissues, respectively) and 1007 cm^−1^ (in the regions: 1023–993 cm^−1^ and 1016–997 cm^−1^ for fixed and unfixed tissues, respectively). For statistical analysis, *t* test was evaluated using commercial software (Origin Pro 9.1, OriginLab). Results are presented as means ± SEM. A difference between means was considered significant if *p* < 0.05.

## Results and discussion

### Raman 3D-AFM imaging of murine aorta

The en face aorta was prepared with the endothelium facing upwards, and thanks to the 3D confocal imaging, a separation of Raman information from different layers was possible. Each time, the measurements were started from the layer where the Raman signal was the most intense, and this plane was designated as *Z* = 0.0 μm. Subsequently, precisely the same area of the vascular wall was re-examined after moving the objective by 0.5 μm up to such a plane where Raman signal was unmeasurably low. In this way, the recorded set of Raman spectra could be assigned to the lower and upper parts of the aorta, corresponding to the media layers of the vascular wall and the endothelium, respectively. This approach offered a unique possibility for the differential analysis of the chemical changes in the endothelium and vascular part of the vessel wall. Nevertheless, it should be added that the information from Raman images may have not originated strictly from one layer of the vessel wall due to variations in endothelial layer thickness and natural roughness of the aorta.

**Fig. 1:**
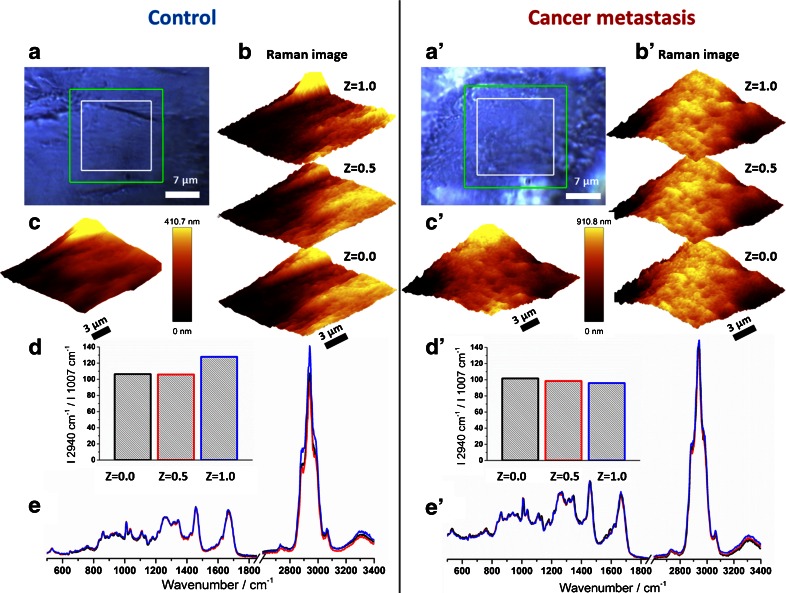
Results of representative measurements of the vessel wall of a control specimen (*left panel*) and a specimen with metastasis (*right panel*) (fixed samples). The areas of Raman and AFM measurements are denoted with *white* and *green rectangles*, respectively, in the visual images (*A*, *A*′). Three Raman distribution images at different depths of the vascular wall were obtained by integration of the band in the range of 2800–3100 cm^−1^ (*B*, *B*′) and compared with the AFM topography (*C*, *C*′). The lipid to protein ratio (*D*, *D*′), defined as the intensity ratio of the band at 2940 cm^−1^ to the band at 1007 cm^−1^, was calculated based on the average spectra (*E*, *E*′) of presented Raman images (*B*, *B*′)

The representative results of two complete measurements of the vascular wall from the control specimen (left panel) and the animal with the cancer metastasis (right panel) are presented in Fig. 1.

Overall, the six and five 3D Raman and AFM measurements were performed for control and metastatic mice, respectively. During the experiment, the flat areas on the aorta surface were selected preferentially (Fig. 1 A and A′) to obtain a set of Raman spectra at different depths of the vessel wall with the signal of the endothelium well separated from the signal of the media layers (Fig. 1 B and B′). To confirm that the surface was truly flat (there was no optical illusion on the visual image), the AFM imaging was performed and the obtained topographic images were compared to the Raman images (Fig. 1 C and C′). Additionally in this case, two times better spatial resolution and incomparably better vertical resolution of AFM compared to Raman imaging revealed the important and valuable surface details. The Raman spectra recorded during the measurement of each layer was averaged to obtain one average Raman spectrum (Fig. 1 E and E′). The lipid to protein ratio was calculated for every average spectrum individually (Fig. 1 D and D′). The overall lipid content was calculated as the ratio of the integral intensity of the band for lipids and proteins (centered at 2940 cm^−1^ and assigned to the C–H stretching vibrations [13]) to the protein marker band (at 1007 cm^−1^, assigned to the ring breathing mode of phenylalanine [14]).

For the control aorta, the apparent difference in the lipid to protein ratio for the endothelium (upper layer, *Z* = 1) relative to the deeper layers is observed. It is evident that the vascular endothelium of the control mice has the higher content of lipids and/or the lower content of the proteins than the deeper layers of the aorta. This effect was not observed in the vascular wall of metastatic animals, where the lipid to protein ratio is comparable for all measured layers. It demonstrates that metastasis causes the endothelium alterations related to the change of lipids and/or protein content.

In order to validate that observed differences between the endothelium of control specimens and animals with metastasis are also measurable in physiological conditions, a similar methodology based on 3D Raman imaging was applied to the unfixed aorta (Electronic Supplementary Material (ESM), Fig. S1).

### Metastasis influence on lipid and protein content in the endothelium

The composition of the split-open murine aorta taken from BALB/c mice at the advance stage of metastasis was compared with that of the aorta taken from age-matched control BALB/c mice. Six weeks after cancer cell transplantation, the number of metastasis in the lung was 45.60 ± 10.26, while in control BALB/c mice the metastatic sites were not present. Metastasis at this stage of disease was associated with robust inflammation and the impairment of nitric oxide (NO)-dependent vasodilation in the aorta [9]. Calculating the integral intensities of marker bands in Raman spectra, it was found that the most prominent differences in the vascular wall of control and metastatic animals were related to the overall lipid and protein content (marker bands at ca. 2940 and 1007 cm^−1^, respectively).

**Fig. 2:**
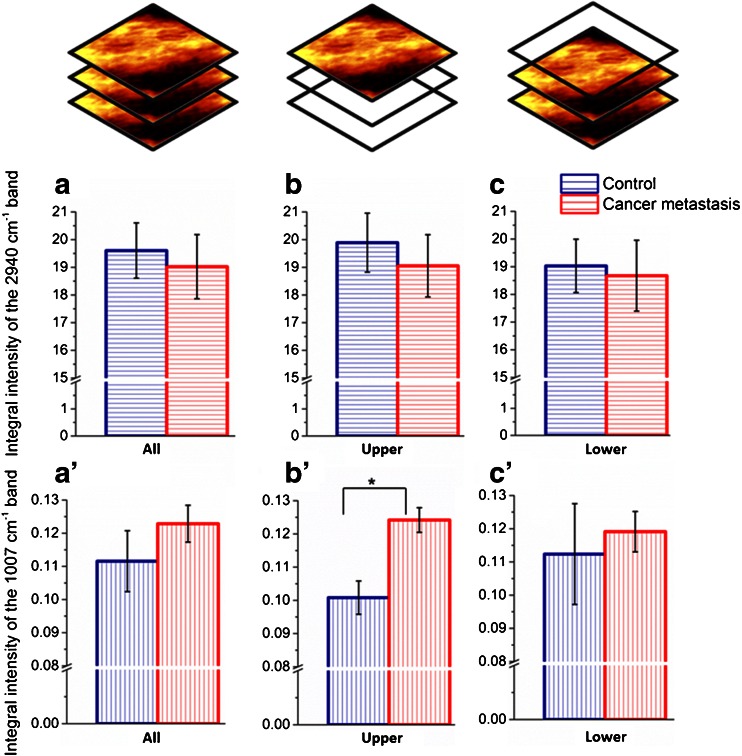
Lipid and protein content in the fixed vascular wall of control and metastatic mice: in the entire volume of the measured vascular wall (*A*, *A*′), in the endothelium (*B*, *B*′) and in the media layer (*C*, *C*′) (*whiskers* denote standard errors, *asterisk* denotes *p*<0.05)

In accordance with the methodology discussed above, obtained Raman images were divided into two groups: upper (referring to the endothelium) and lower ones (referring to the media layers). The complete analysis was performed individually for each group in order to verify in which parts of the vascular wall alterations due to the pathology took place. The results showing changes in the entire volume of the vascular wall (all) and selectively in the endothelium (upper) and media layers (lower) for fixed tissues are presented in Fig 2. The results demonstrated similar trends for both fixed (Fig. 2) and unfixed (ESM Fig. S2) samples of the vessel wall.

The simultaneous slight decrease of the overall lipid content and the significant increase in the protein level were observed in the vascular wall (Fig. 2 A and A′). Both changes were definitely limited to the endothelium layer (Fig. 2 B and B′), while the lipid and protein content was practically unchanged in the media layers for the studied group of mice (Fig. 2 C and C′). It is clear that a specific response from the endothelium was so significant (Fig. 2 B and B′) that it strongly affected the results averaged over the whole measured content (Fig. 2 A and A′). Nevertheless, this 3D Raman imaging approach clearly demonstrated that the disturbances in the lipid and, particularly, protein level can be treated as a specific endothelium response and a biomarker of endothelial dysfunction upon cancer metastasis.

In the measured volume of the vessel wall, the lipid content insignificantly decreased for the metastatic mice relatively to the control (Fig. 2(A)). Nevertheless, for the endothelium, this effect was more pronounced i.e., the 4 % decrease in the lipid content was observed for specimens with metastasis (Fig. 2(B)). A considerably more striking effect of the metastasis was the overproduction of proteins. The intensity of the band at 1007 cm^−1^ increased up to 18 % in the endothelium of metastatic mice in comparison with the control (Fig. 2(B′)).

Also for the unfixed aorta, endothelium was the layer primarily involved in the response to the cancer metastasis (ESM Fig. S2). It is clear that the lipid level decreased (7 %) and the protein content significantly increased (approx. 12 %, *p* = 0.034) upon pathology development, compared to the control. Although the trend was similar if the whole volume of the sample was considered, no statistically significant differences between the vascular wall of the BALB/c mice at the advance stage of metastasis and control were observed.

### Spectral characteristics of metastasis-induced changes in endothelium

**Fig. 3:**
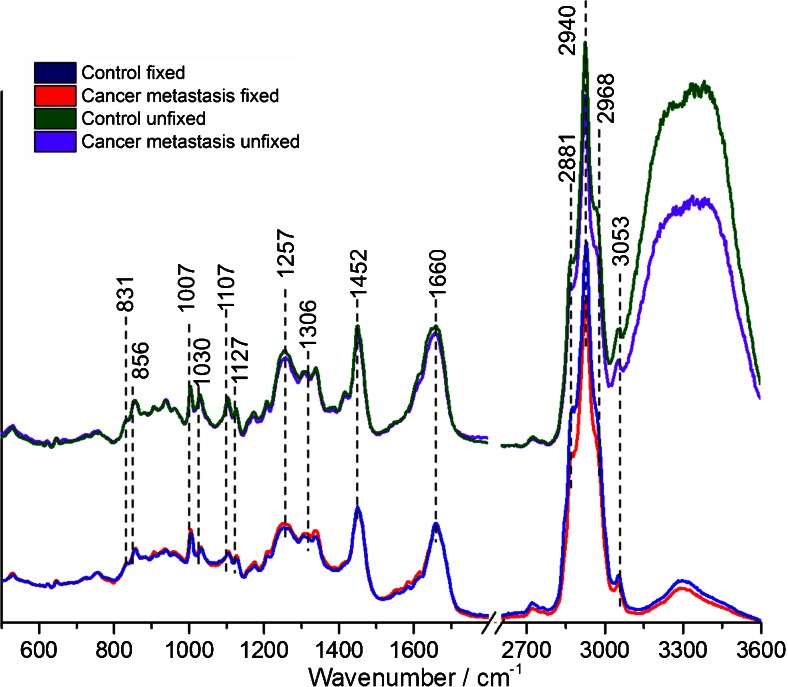
Characteristics of Raman average spectra of endothelium in the fixed and unfixed vascular wall. The comparison of the average spectra of the endothelium: control fixed (*blue*), metastasis-altered fixed (*red*), control unfixed (*green*), and metastasis-altered unfixed (*violet*) mice. Spectra were normalized to the phenylalanine band at 1007 cm^−1^

Differences in the average spectral profiles of the control and metastasis-altered endothelium both in fresh and fixed samples are presented in Fig. 3.

The most prominent differences in the Raman spectra of control and metastasis-changed endothelium were noticeable in the high wavenumber range (2800–3100 cm^−1^), where the observed broad band at ca. 2940 cm^−1^ is a superposition of features due to the stretching vibrations of various lipid and protein CH_2_ and CH_3_ groups. The bands at ca. 2968 and 2940 cm^−1^ originate from the CH_3_ asymmetric and symmetric stretching vibrations, respectively, for both proteins and lipids [13]. The characteristic signal for lipids is a shoulder at 2881 cm^−1^ [13, 15]. The decrease of the intensity of the band in the 2800–3100 cm^−1^ region (both for fixed and unfixed samples) represents the overall decrease in the lipid to protein ratio in the endothelium, as spectra were previously normalized to the phenylalanine band. The band at 3053 cm^−1^ corresponds to the C–H stretching vibrations observed in aromatic amino acids (phenylalanine, tryptophan) [16]. In the analyzed spectra, there is no contributions from the =CH stretching modes, typically exhibited as the band at ca. 3005 cm^−1^ and distinctive for unsaturated lipids [13]. Although the substantial biochemical information on proteins is usually connected to the band located at 1660 cm^−1^, i.e., the amide I [14], it is obvious that this band is also related to lipids and assigned to the C=C stretching vibrations of unsaturated triacylglycerols, cholesterols, and membrane lipids [13]. Nevertheless, the lack of the band at 3005 cm^−1^ indicates a marginal contribution of unsaturated lipids into the amide I band. For the unfixed aorta, the analysis of the amide I band is even more complicated, due to water contribution. Both effects, broadening of the amide I band and the presence of the very intense featureless band at ca. 3300 cm^−1^ for spectra of the unfixed aorta, resulted from water environment. The band at around 1452 cm^−1^ is attributed to the CH_2_ wagging modes and contains overlapped signals from lipids [13] and proteins [14].

## Conclusions

Alterations in the vascular wall of the aorta in the murine model of metastatic breast cancer (4T1) were studied with the help of 3D Raman-AFM imaging of the endothelium. We introduced a novel methodology to study a vascular wall and identified that the chemical changes in the aorta taken from mice at the stage of advance metastasis related mostly to the increase of the protein content accompanied by the decrease of the lipid content. Our approach was based on the 3D confocal imaging of the vessel wall and enabled the analysis of the endothelial and vascular muscle layers of the aorta individually. Application of this approach resulted in the conclusion that observed chemical changes were limited to the endothelium.

Importantly, in the same experimental model of the murine metastatic breast cancer (4T1), we reported that tumor progression and development of metastasis resulted in the impairment of NO-dependent function in the aorta that was most likely due to the robust cancer-associated inflammation [9]. Accordingly, in the present work, using the same model, for the first time we characterized the biochemical correlates of the endothelial dysfunction associated with cancer metastasis. Here, we identified that the increase of the protein content accompanied by the slight decrease of the lipid content is the prominent feature of cancer metastasis-induced peripheral endothelial dysfunction. The fall in lipids is difficult to explain, although cellular endothelial injury of any kind is associated with a fall of lipid content. The increased content of proteins, however, may be related to the activation of pro-inflammatory and pro-thrombotic endothelial phenotype. Indeed, in the advanced stage of cancer progression (6th week after cancer cell inoculation), the impairment of NO-dependent response in the aorta was associated with the upregulation of vascular COX-2-derived PGI_2_ and increased expression of von Willebrand factor, vWF [9]. Furthermore, various pro-inflammatory pathways can be activated in the dysfunctional state of the endothelium when NO production is impaired including VCAM-1, ICAM-1, other adhesion molecules, MCP-1, and other chemokines and many other pro-inflammatory molecules. Interestingly, the phenotype of “cancer-associated” dysfunctional endothelial cells (DECs) was characterized in vitro [2] showing that the expression of many pro-inflammatory NF-ĸB target genes was significantly increased while the expression of quiescence-promoting, anti-inflammatory genes was decreased. DECs seemed to be much more active, e.g., secreted much larger amounts of proteins [2]. Taking all these data together, we suggest that changes in the protein content of endothelial cells, observed in Raman spectra, result from dysfunctional activation of the endothelium related to cancer and ongoing inflammation. Coincidently, the biochemical changes in the endothelium, i.e., the increase of the protein and the decrease of the lipid content upon cancer metastasis, are exactly the same as the alterations in the tissue upon its transformation into breast tumor itself [17]. What is the pathophysiological meaning of this fact remains to be elucidated.

## Electronic supplementary material

Below is the link to the electronic supplementary material.

ESM 1 (PDF 991 kb)
