# Supplementary material for: 3D Raman imaging of systemic endothelial dysfunction in the murine model of metastatic breast cancer
Source: Anal Bioanal Chem. 2016 Mar 2;408:3381–7. doi: 10.1007/s00216-016-9436-9 (PMC4837206; doi:10.1007/s00216-016-9436-9)
Supplement: Supplementary file 1 — (PDF 991 kb) [file 216_2016_9436_MOESM1_ESM.pdf]

Analytical and Bioanalytical Chemistry

Electronic Supplementary information

3D Raman imaging of systemic endothelial dysfunction in the murine model of metastatic breast cancer

Marta Z. Pacia, Elżbieta Buczek, Agnieszka Blazejczyk, Aleksandra Gregorius, Joanna Wietrzyk, Stefan Chlopicki, Malgorzata Baranska, Agnieszka Kaczor

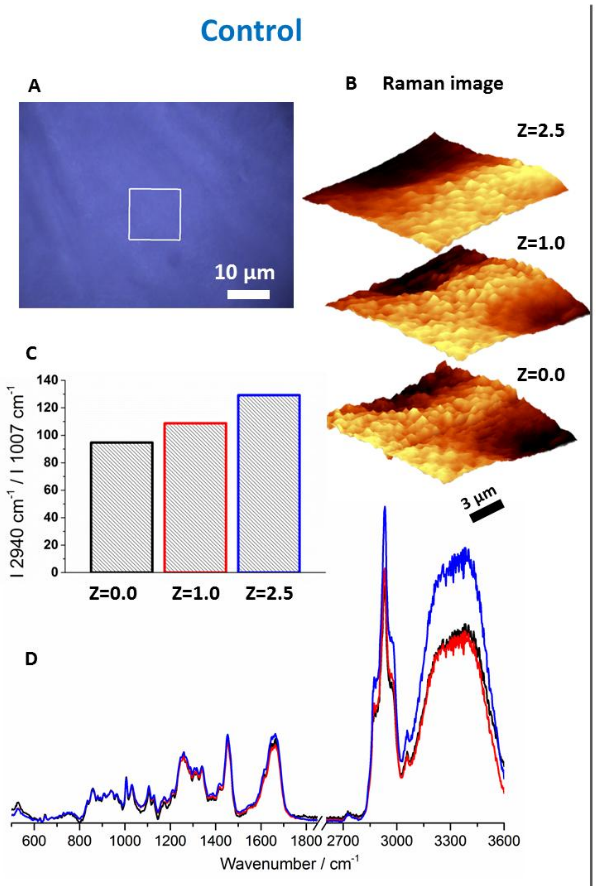

Fig. S1 Results of representative measurements of tissues the vessel wall in control (left panel) and upon cancer metastasis (right panel) (unfixed samples). The areas of Raman measurements are denoted with white and green rectangles, respectively, in the visual images (A, A'). Three Raman distribution images at different depths of tissue were obtained by integration of the band in the range of 2800-3100 cm^{-1} (B, B'). The lipid to protein ratio (C, C'), defined as the intensity ratio of the band at 2940 cm^{-1} to the band at 1007 cm^{-1}, was calculated based on the average spectra (D, D') of presented Raman images (B, B')

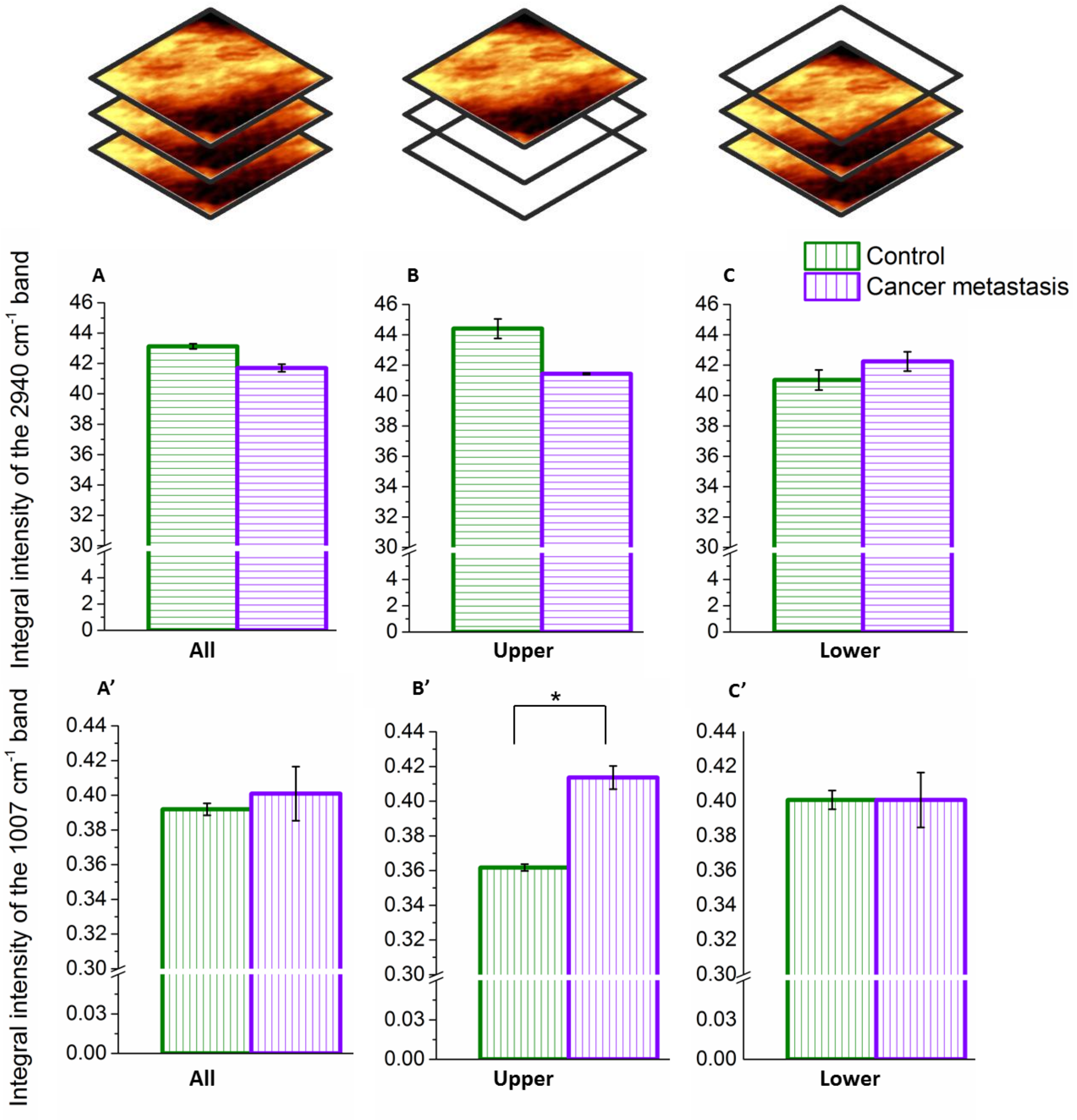

Fig. S2 Lipid and protein content in unfixed tissues of control and metastatic mice. The level in lipid and protein content: in the entire volume of measured tissue (A, A'), in the endothelium (B, B') and in media layer (C, C') (whiskers denote standard errors)
